# Number and Distribution of Mouse Retinal Cone Photoreceptors: Differences between an Albino (Swiss) and a Pigmented (C57/BL6) Strain

**DOI:** 10.1371/journal.pone.0102392

**Published:** 2014-07-16

**Authors:** Arturo Ortín-Martínez, Francisco M. Nadal-Nicolás, Manuel Jiménez-López, Juan J. Alburquerque-Béjar, Leticia Nieto-López, Diego García-Ayuso, Maria P. Villegas-Pérez, Manuel Vidal-Sanz, Marta Agudo-Barriuso

**Affiliations:** 1 Instituto Murciano de Investigación Biosanitaria-Virgen de la Arrixaca, El Palmar, Murcia, Spain; 2 Departamento de Oftalmología, Facultad de Medicina, Universidad de Murcia, Espinardo, Murcia, Spain; Hanson Institute, Australia

## Abstract

We purpose here to analyze and compare the population and topography of cone photoreceptors in two mouse strains using automated routines, and to design a method of retinal sampling for their accurate manual quantification. In whole-mounted retinas from pigmented C57/BL6 and albino Swiss mice, the longwave-sensitive (L) and the shortwave-sensitive (S) opsins were immunodetected to analyze the population of each cone type. In another group of retinas both opsins were detected with the same fluorophore to quantify all cones. In a third set of retinas, L-opsin and Brn3a were immunodetected to determine whether L-opsin^+^cones and retinal ganglion cells (RGCs) have a parallel distribution. Cones and RGCs were automatically quantified and their topography illustrated with isodensity maps. Our results show that pigmented mice have a significantly higher number of total cones (all-cones) and of L-opsin^+^cones than albinos which, in turn, have a higher population of S-opsin^+^cones. In pigmented animals 40% of cones are dual (cones that express both opsins), 34% genuine-L (cones that only express the L-opsin), and 26% genuine-S (cones that only express the S-opsin). In albinos, 23% of cones are genuine-S and the proportion of dual cones increases to 76% at the expense of genuine-L cones. In both strains, L-opsin^+^cones are denser in the central than peripheral retina, and all-cones density increases dorso-ventrally. In pigmented animals S-opsin^+^cones are scarce in the dorsal retina and very numerous in the ventral retina, being densest in its nasal aspect. In albinos, S-opsin^+^cones are abundant in the dorsal retina, although their highest densities are also ventral. Based on the densities of each cone population, we propose a sampling method to manually quantify and infer their total population. In conclusion, these data provide the basis to study cone degeneration and its prevention in pathologic conditions.

## Introduction

In the mammalian retina, cone photoreceptors receive and transduce the spectral information of the environment. In the great majority of non-primate mammals there are two spectral cone types, each carrying a different visual pigment or opsin. One opsin is sensitive to short-wavelengths (S-opsin), and the other to middle-to-long-wavelengths (L-opsin, also referred as M-opsin; here we have used L-opsin because it responds to the longest wavelenght in rodents). In the mouse, rat and other murid rodents, the peak sensitivity of the S-pigment is in the ultraviolet range [1]–[3].

As a rule, when a cone expresses only one type of opsin it is considered a genuine S or genuine L cone. The concept of genuine cones being those expressing only one opsin is strengthened by their circuitry. In dichromatic mammals, bipolar cells receive cone input in three mutually exclusive ways: from cones expressing S-opsin, but not detectable amounts of L-opsin (genuine-S cones), from cones expressing only L-opsin (genuine-L cones), and from cones that express both opsins (dual cones) [4], [5]. Dual cones [6] are found in several species [7] and they are very abundant in the mouse retina, where the majority of cones co-express both opsins [2], [4], [8], [9]. Opsin co-expression though, does not prevent mice from colour discrimination [1], [10].

Opsins are expressed in the outer segment of photoreceptors, and they are definitive markers of cell type. Each cone type can be detected using anti-S- or anti-L- opsin antibodies [9], [11], and the entire cone population can be identified by using either lectins (peanut agglutinin) [9] or by visualizing both primary antibodies with the same fluorophore [12], [13]. To quantify cones, most laboratories resort to retinal sampling and manual quantification, providing the results as densities or extrapolated total numbers.

Cone distribution in the mouse is not homogeneous [2], [8], [11], [14]–[18]. Indeed, cone topography in the common pigmented mice is quite striking [19]: cones that express the S-opsin (S-opsin^+^cones) are very sparse in the dorsal retina and very abundant in the ventral retina, while cones that express the L-opsin (L-opsin^+^cones) are evenly distributed across the retina. But because L-opsin protein levels are lower in the ventral retina [8], [15], in pigmented mice cone fields are complementary [16]–[18].

Another important issue are the genetic variations among mouse strains [11] that induce, among other phenotypic outcomes, changes in the topography of S-opsin^+^cones in such a way that in albino mice, these are abundant in the dorsal retina [8], [11]. The same difference between albino and pigmented animals has been recently reported in another rodent species, the deer mouse [20].

The house mouse (*Mus musculus*) is an essential animal model for research. Even though mice have a poor visual acuity compared to primates, they are broadly used to understand how vision works [21]. Besides, there are many mouse mutants that mimic human retinal degenerations caused by photoreceptor death [22]. To study the course of photoreceptor loss and/or the potential protective effects of a given treatment, the better the knowledge about their number and distribution, the more accurate the analyses.

By automatically counting mouse and rat retinal ganglion cells (RGCs) and rat cones, our group has reported their normal number and distribution [12], [23]–[28], and investigated their fate in pathological models, alone or after neuroprotective therapies [23], [25], [26], [29]–[34].

Despite the number of groups working on cones in mouse there is not, to date, a thorough study of their total numbers and topography, nor has the proportion of dual and single pigment cones been fully addressed. Thus, based on our expertise, we have used bespoken state-of-the-art automated routines to quantify all cones and cones that express the S- or the L- opsins in mouse; with these data the number of genuine or dual cones was calculated. The second objective was to determine their detailed topography using high resolution isodensity maps. Finally, by analyzing these isodensity maps we have selected sampling areas to accurately quantify and infer each cone population when automated routines are not available. All these analyses were carried out in two inbred house mouse strains commonly used in research, one pigmented (C57/BL6) and one albino (Swiss). These data provide the necessary background information to study cone degeneration in albino and pigmented mouse models of human degenerative retinal diseases. Parts of this work have been presented in abstract form [35]–[36].

## Materials and Methods

### Ethic statement and animal handling

Four months old female albino Swiss and pigmented C57/BL6 mice were obtained from the University of Murcia breeding colony. Animal handling was carried out in accordance with the Association for Research in Vision and Ophthalmology and European Union guidelines for the use of animals in research. This study was approved by the Ethics Committee for Animal Research of the University of Murcia and University Hospital Virgen de la Arrixaca (Comité Ético de Experimentación Animal (CEEA) del Hospital Universitario Virgen de la Arrixaca, Murcia, Spain). All animals were sacrificed with an i.p. injection of an overdose of pentobarbital (Dolethal, Vetoquinol, EspecialidadesVeterinarias, S.A., Alcobendas, Madrid, Spain).

### Animal groups

Animals were divided according to the immunodetection protocol:

Double detection of S- and L- opsins. Each opsin was detected with a different fluorophore. These retinas served to quantify the whole population of S- and L- opsin positive cones (n = 10 retinas pigmented and n = 8 albino).

Detection of S- and L- opsins with the same fluorophore. In these retinas the whole population of cones were quantified (henceforth “all-cones”) (n = 10 retinas pigmented and n = 8 albino).

Double detection of Brn3a and L-opsin. These retinas were used to assess whether RGCs and L-cones have a parallel distribution, as described for rat [12] (n = 4 retinas albino and n = 6 pigmented).

### Retinal dissection

Unless otherwise stated, all the reagents were from Sigma-Aldrich, (Alcobendas, Madrid, Spain).

Animals were perfused transcardially with 4% paraformaldehyde (PFA) in phosphate buffer 0.1 M after a saline rinse.

Right after deep anaesthesia and before fixation a suture was placed on the dorsal pole of each eye. Retinas were dissected as flattened whole-mounts by making four radial cuts (the deepest one in the dorsal pole), post-fixed for an additional hour in 4%PFA and kept in phosphate buffered saline (PBS) till further processing.

### Immunohistofluoresence

Immunodetection was carried out using standard procedures in our group. First, retinas were permeated in PBS 0.5% Triton X-100 (Tx) by freezing them during 15 minutes at −70°C. In our experience, this step is essential for the proper penetration of the antibody in the tissue and, thus, for getting a consistent immunodetection. In spite of the low temperature and the lack of cryoprotectant, there is no visible tissue damage, at least at our analysis level. After the freezing step, retinas were thawed at room temperature, rinsed in new PBS-0.5%Tx and incubated overnight at 4°C with the appropriate mixture of primary antibodies diluted in blocking buffer (PBS, 2% normal donkey serum, 2%Tx). Retinas were washed three times in PBS and incubated 2 hours at room temperature with the secondary antibodies diluted in PBS-2%Tx. Finally, retinas were thoroughly washed in PBS-0.5%Tx and, after a last rinse in PBS, mounted scleral side up on subbed slides and covered with anti-fading solution (Vectashield Mounting Medium, Vector Laboratories). Retinas immunodetected for Brn3a and L-opsin were mounted between two cover-slips that were attached to a slide with cellophane. This allowed flip-flopping the retinas to photograph first the L-cones (scleral side) and then the RGCs (vitreal side).

### Antibodies and working dilutions

Primary antibodies: S-opsin∶goat anti-OPN1SW, 1∶1000 (sc-14363, Santa-Cruz Biotechnologies, Heidelberg, Germany), L-opsin∶rabbit anti-opsin red/green, 1∶1200 (ab5405, Millipore Ibérica, Madrid, Spain), Brn3a∶goat anti-Brn3a, 1∶750 (sc-31984, Santa-Cruz Biotechnologies, Heidelberg, Germany).

Secondary antibodies: donkey anti-goat Alexa Fluor 488, donkey anti-rabbit Alexa Fluor 488 and donkey anti-rabbit Alexa Fluor 594 (Jackson ImmunoResearch, Suffolk, UK), all used at 1∶500.

### Image acquisition

To make reconstructions of retinal whole-mounts, these were photographed with a ×20 objective under an epifluorescence microscope (Axioscop 2 Plus; Zeiss Mikroskopie, Jena, Germany) equipped with rhodamine (BP546/12, LP 590) and fluorescein (BP 450/490, LP 515/565) filtres, and a computer-driven motorized stage (ProScan H128 Series; Prior Scientific Instruments, Cambridge, UK) controlled by Image-Pro Plus (IPP 5.1 for Windows; Media Cybernetics, Silver Spring, MD, USA) as previously described [23], [28], [29]. Individual frames were tiled to reconstruct the whole-mounts (140 individual frames/retina). To discard possible fluorescence leaking from the fluoresceine (green) to the rhodamine (red) filter and vice versa, in a previous experiment retinas were single immunodetected and the acquisition settings forced (high exposure time). When testing the alexa-488 there was no signal in the red filter and conversely, the alexa-594 signal was not observed in the green filter.

### Image processing: automated quantification

We developed two automated counting routines using the IPP software macro language, one to quantify the S-opsin^+^cones and all-cones, and the other to quantify the L-opsin^+^cones. These routines counted positive cells in each of the 140 frames spanning a retina.

#### S-opsin^+^cones and all-cones

First, images were converted to 16-bit gray scale to discard colour information, followed by the application of the *higauss* enhancement filter. The resulting image was then filtered through a large spectral filter *edge+*, which extracts positive edges from the dark background.

#### L-opsin^+^cones

First a flatten filter was applied to eliminate luminosity variations in the image. Next, images were converted to 16-bit gray scale to discard colour information. After that a best fit was performed to enhance positive objects, followed by the higauss enhancement filter. The resulting image was then filtered with a large spectral filter edge+, to extract positive edges from the dark background.

Cones were counted within predetermined parameters to exclude objects that were too large or too small to be outer segments. Once counting routines were performed, data of each count were displayed and exported by dynamic data exchange to a spreadsheet (Microsoft Office Excel 2003; Microsoft Corp.), and the data were filed and saved for further analysis.

Brn3a^+^RGCs were automatically quantified as previously described [25], [26].

### Validation of the automated counting methods

Three different experienced investigators counted manually, in a masked fashion, a total of 20,112 L-opsin^+^cones, 13,036 S-opsin^+^cones and 43,197 cones (S+L, all-cones). These images represented different density regions and were randomly selected from five whole-mounted retinas. In total 8 to 15 images per marker were counted. The automated quantification of these same images resulted in 18,799, 13,012, and 42,969 L-opsin^+^cones, S-opsin^+^cones, and all-cones, respectively. Finally, each automated method was statistically compared to its manual counterpart (SigmaStat for Windows version 3.11; Systat Software, Inc., Richmond, CA). The Pearson correlation coefficient between the automated routine and the manual quantification was 0.97 (R^2^ = 0.95), 0.98 (R^2^ = 0.97) and 0.92 (R^2^ = 0.84) for the L-opsin^+^cones, S-opsin^+^cones, and all-cones counts, respectively. Thus, the automated routines were valid and reliable.

### Isodensity maps

Topographical distribution of L-opsin^+^cones, S-opsin^+^cones, and all-cones was demonstrated with isodensity maps. These maps are filled contour plots generated by assigning to each one of the 25 subdivisions (areas of interest, 0.0087 mm^2^) of each capture frame (20× objective, 0.2186 mm^2^) a colour code according to its cone density, within a 8-step colour-scale ranging from 0 (purple) to ≥16,000 L-opsin^+^cones/mm^2^; ≥18,000 S-opsin^+^cones/mm^2^, and ≥20,000 (pigmented) or 18,000 (albino) all-cones/mm^2^ (red).

RGC isodensity maps were generated as previously described [25], [26].

### Statistical analysis

Statistical comparisons were carried out using the SigmaStat for Windows Version 3.11 program (Systat Software, Inc., Richmond, CA). Data are presented as mean ± standard deviation. Differences were considered significant when p<0.05 and tests are detailed in results.

## Results

### Opsin immunodetection

**Figure 1:**
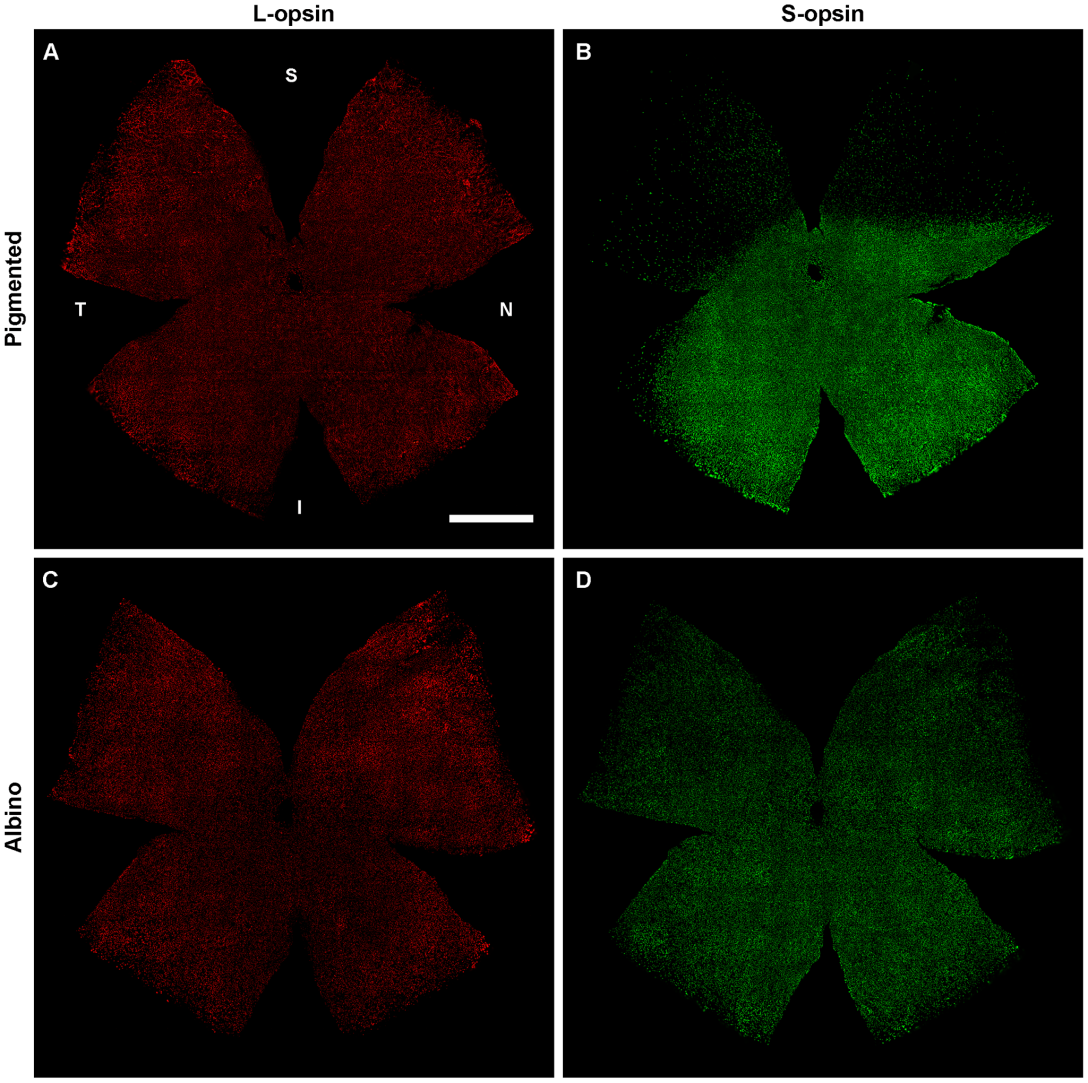
Opsin immunodetection in pigmented (C57/BL6) and albino (Swiss) mouse strains. Flat mounted left retinas from a pigmented (**A,B**) and an albino (**C,D**) mouse where the L- and the S- opsin have been double immunodetected. S: superior, N: nasal, I: inferior, T: temporal. Bar: 1 mm.

**Figure 2:**
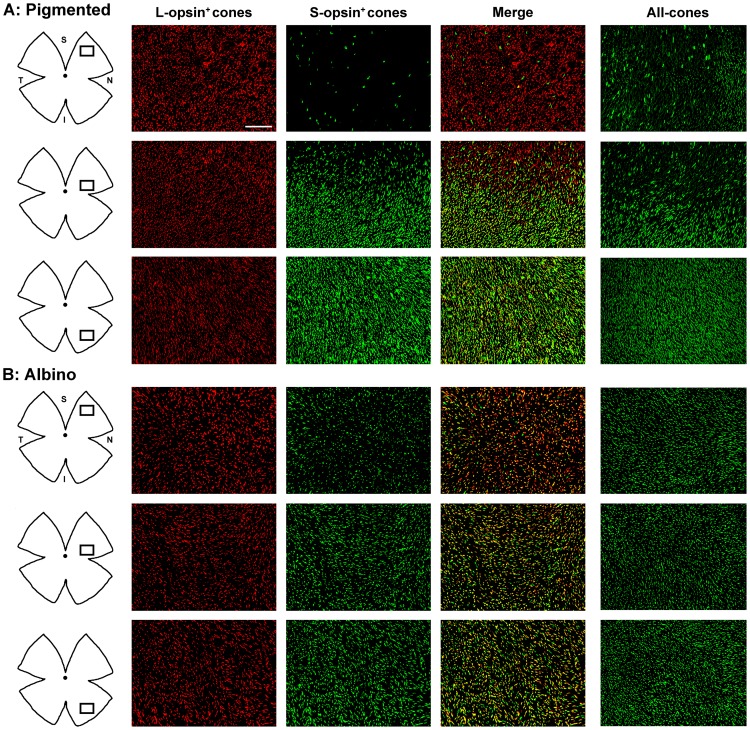
Opsin expression in pigmented and albino mice. Magnifications from pigmented (**A**) and albino (**B**) mice flat mounted retinas showing L-opsin^+^cones, S-opsin^+^cones and their merged image. The rightmost column shows all-cones (both opsins developed with the same fluorophore). In the retinal drawings on the left, is shown the area where the magnifications were taken from. S-opsin^+^cones are sparse in the superior retina of the pigmented strain and abundant in the albino one. In both strains most genuine S-cones are found in the inferior retina. In the pigmented strain majority of genuine L-cones lay in the superior retina, while in the albino mouse majority of L-cones are dual. And so dual cones are found across the retina in the albino strain and mostly restricted to the ventral retina in the pigmented one. S: superior, N: nasal, I: inferior, T: temporal. Bar: 100 µm.

Here we have identified and quantified cones by immunodetection of their outer segment. This immunodetection relies on the removal of the retinal pigmented epithelium (RPE). This step is delicate, as part of the outer segment of the photoreceptors may be pulled out with the RPE, thus creating spots bare of outer segments. In our hands, most of the retinas were successfully dissected (see **figures 1** and **2**), however few of them were not and, therefore, were not included in this study.

In one set of retinas each opsin was identified with a different fluorophore to analyze the population of L-opsin^+^ and S-opsin^+^ cones (**Figure 1** and **2**). In another set of retinas, both opsins were visualized with the same fluorophore to study the whole cone population (all-cones) irrespectively of their opsin expression (**Figure 2**, right column). As shown in both figures, the L-opsin is expressed across the retina in both mouse strains. However, the S-opsin pattern remarkably differs between them. While in the C57/BL6 pigmented strain there is a gradient of S-opsin from few positive cones in the superior retina to a high number of them in the ventral retina (**Figure 1B**), as reported [8], [16], [17], in the albino Swiss strain, the S-opsin signal looks homogeneous across the retina. This is detailed in the topographic analysis below.

Magnifications in **figure 2** (compare “L-opsin^+^cones” and “S-opsin^+^cones” columns with “merge” column) suggest that: i/ genuine S-cones are found preferentially in the ventral retina in both strains (green signal); ii/ genuine L-cones are (preferentially) located in the dorsal retina in pigmented mice (red signal); iii/ in the albino strain most L-cones are dual and the few genuine L-cones are scattered; iv/ in both strains there is a high level of opsin co-expression (yellow signal), and v/ dual cones are found across the retina in albino animals, while in pigmented ones are mostly confined to the ventral retina.

### Total number of cones

Next step was to develop customized automated methods to quantify the total number of cones in the sets of retinas abovementioned; results are shown in **Table 1**. These quantitative data show first that in the pigmented strain the number of cones expressing the L-opsin is significantly higher than that of those expressing the S-opsin, being the inverse in the albino mouse. Second, that the pigmented strain has a significantly higher number of total cones and L-opsin^+^cones than the albino. In turn, the albino mouse has a significantly higher population of S-opsin^+^cones. And third, that in both strains the total number of cones is smaller than the sum of the L- and S- opsin^+^cones. This result quantitatively supports the elevated opsin co-expression observed in mouse.

**Table 1:** Total number of cones in the mouse retina.

Pigmented (C57/BL6)					Albino (Swiss)				
Automated quantification of cones immunodetected with					Automated quantification of cones immunodetected with				
*Retina*	L-opsin	S-opsin	*Retina*	Both opsins	*Retina*	L-opsin	S-opsin	*Retina*	Both opsins
*1RR*	134,873	124,910	*6RR*	180,029	*1RR*	126,928	162,086	*5RR*	155,961
*1LR*	138,559	126,628	*6LR*	179,124	*1LR*	128,745	162,961	*5LR*	154,754
*2RR*	146,677	123,338	*7RR*	183,560	*2RR*	130,982	174,448	*6RR*	160,678
*2LR*	150,500	132,462	*7LR*	191,131	*2LR*	136,576	165,345	*6LR*	152,856
*3RR*	127,004	118,677	*8RR*	178,221	*3RR*	113,030	138,824	*7RR*	161,465
*3LR*	133,058	126,799	*8LR*	189,760	*3LR*	121,106	149,470	*7LR*	156,738
*4RR*	127,932	108,559	*9RR*	190,846	*4RR*	90,292	111,108	*8RR*	146,182
*4LR*	139,928	118,219	*9LR*	179,852	*4LR*	91,729	109,217	*8LR*	135,707
*5RR*	129,028	108,828	*10RR*	181,472					
*5LR*	123,992	107,744	*10LR*	172,010					
**Mean number**	**135,155^§^**	**119,616^#^**		182,601*		**117,424^§^**	**146,682^#^**		153,043*
**SD**	**8,742**	**8,756**		**6,252**		**17,721**	**24,958**		**8,468**
**Area (mm^2^)**	**14.4±0.7**			**15.0±0.5**		**15.1±0.6**			**15.0±1**
Mean±SD									
**Density**									
(cones/mm^2^)									
Mean	**9,420**	**8,325**		**12,171**		**7,790**	**9,730**		**10,251**
SD	**599**	**387**		**430**		**1,391**	**1,881**		**989**
Max	**14,610**	**20,466**		**18,157**		**13,879**	**17,553**		**16,552**
Min	**3,652**	**213**		**6,808**		**4,337**	**5,485**		**4,138**

Total number of cones quantified in individual retinas and their mean number ± standard deviation (SD). **L-opsin:** number of cones that express the L-opsin (L-opsin^+^cones: genuine-L and dual cones); **S-opsin:** number of cones that express the S-opsin (S-opsin^+^cones: genuine-S and dual cones). **Both opsins:** quantification of all-cones after double immunodetection of S- and L-opsins with the same fluorophore (all-cones: genuine-L, genuine-S, and dual cones). Last rows show the retinal area and the mean cone density across the retina. In addition are shown the maximum (max) and minimum (min) densities found. These values were extracted from the intense scrutiny carried out during the sampling method analysis (see figure 6), were samples of 0.05 mm^2^ were independently quantified. LR: left retina, RR: right retina. There are significantly more L-opsin^+^cones and all-cones in the pigmented than in the albino strain (Ttest, **^§^**p = 0.006, *p≤0.001, respectively) while for the S-opsin^+^cones is the reverse (**^#^**p = 0.01).

Among the analyzed animals, there was one albino (number 4), whose number of cones was much lower than in the rest, thus increasing the variability (see the SD for the L- and S- opsin^+^ cones). Because in this work we purpose to study the normality of the cone population in mice commonly used in research, we decided to include it since it shows the inter-individual variability, even among animals from the same highly inbred strain.

Retinal area was also measured to calculate the mean cone density. However, mean densities should be taken with caution as the distribution of cones is not homogeneous across the retina. In fact, there is a wide range of cone density (**Table 1**, last row) with a ratio between highly dense/sparsely dense regions from 2.6 (all-cones, pigmented strain) to 96 (S-opsin^+^cones, pigmented strain).

**Figure 3:**
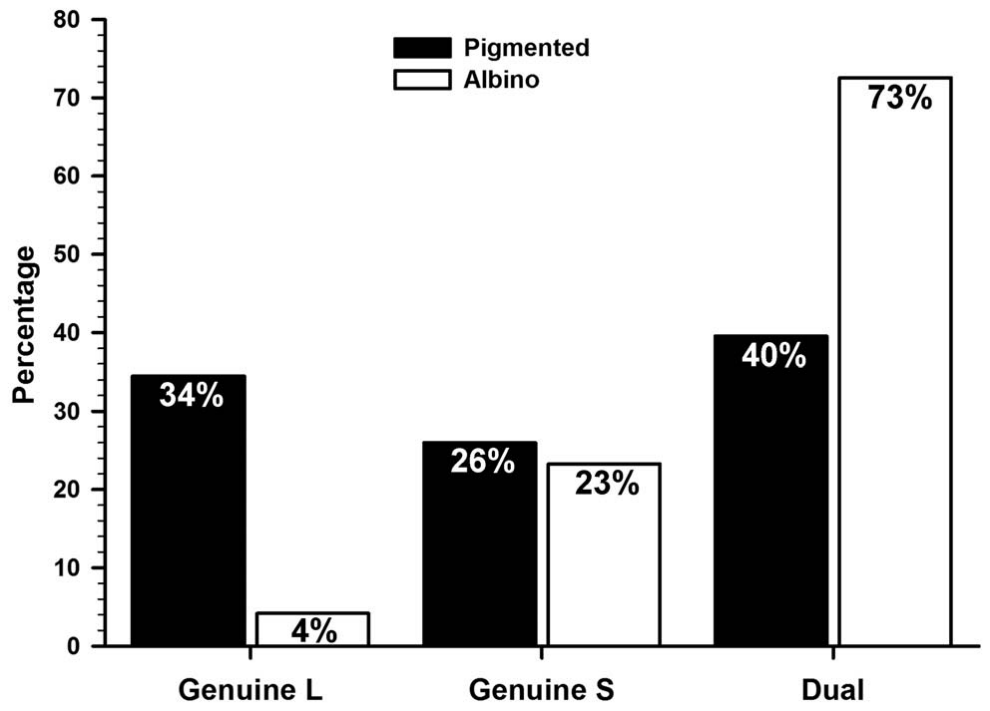
Percentage of genuine and dual cones in albino and pigmented mice. Graph showing the percentage of genuine-L, genuine-S and dual cones in both strains. Percentages were calculated based on data from table 2 and considering 100% the total number of cones (Table 1, all-cones -both opsins-).

Finally, using the total mean numbers of L-opsin^+^cones, S-opsin^+^cones and all-cones, we calculated the number of genuine L, genuine S and dual cones in both strains (**Table 2**). These numbers indicate that in the pigmented mouse approximately 26% of cones are genuine L, 34% genuine S, and the remaining 40% dual cones, while in the albino strain about 73% of cones are dual, 23% are genuine S, and 4% are genuine L (**Figure 3**). Therefore, to quantify the whole cone population in the pigmented strain both opsins should be detected whereas in the albino strain immunodetection of the S-opsin suffices, because 96% of its cones express it.

**Table 2:** Number of genuine and dual cones in the mouse retina.

	Genuine L	Genuine S	Dual
	*Both−Sopsin*	*Both−Lopsin*	*Both−(genuineS*+*genuineL)*
**Pigmented**	62,984	47,445	72,171
**Albino**	6,360	35,619	111,063

With data from table 1, the population of genuine L, genuine S, and dual cones was inferred (see column headlines).

### Cone distribution

**Figure 4:**
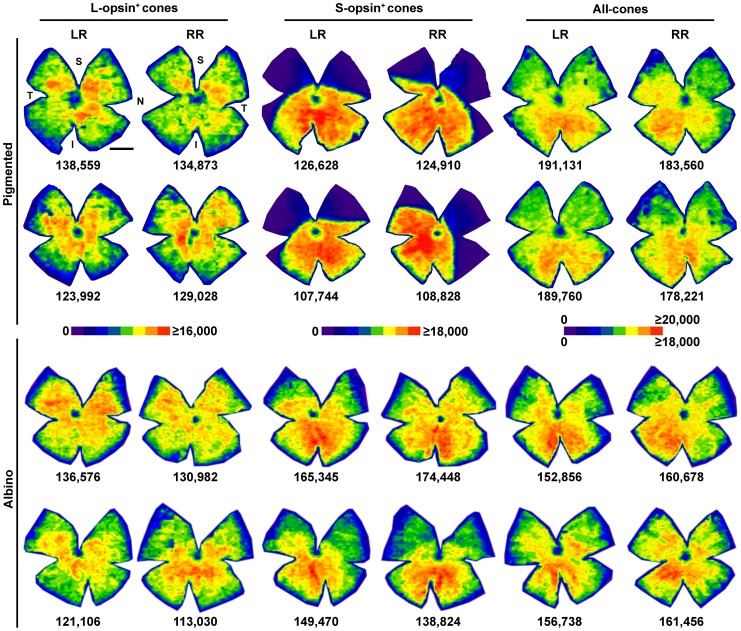
Cone distribution in the mouse retina. Isodensity maps showing the topography of cones in pigmented and albino mice. For each population and strain two left and two right retinas are shown. Notice that because L-opsin^+^ and S-opsin^+^ cones were double immunodetected the first and third, and the second and fourth map in each row are from the same retinas. All-cones maps were generated from another set of retinas in which both α-opsin antibodies were visualized using secondary antibodies coupled with the same fluorophore, hence all cones were taken into account irrespectively of their opsin expression. Below each map is shown the number of cones quantified in its corresponding retina. The colour scale density code is placed in the middle of the figure. LR: left retina, RR: right retina. S: superior, N: nasal, I: inferior, T: temporal. Bar: 1 mm.

Quantitative data gathered from flat mounts can be transferred to a graphical representation of retinal density. To increase the definition and accuracy of these colour contour plots, or isodensity maps, each microscopic frame (0.2186 mm^2^) is divided into 25 areas of interest (0.0087 mm^2^) and within each one, cone density is calculated. Densities are then translated into colours, using an eight colour scale that goes from 0 cones/mm^2^ (purple) to a maximum (red) that was set according to each population (**Figure 4**).

The topography of L-opsin^+^cones is similar in both strains, they are denser around the optic nerve, and their density gradually decreases from the centre to the periphery.

The distribution of S-opsin^+^cones is very different among strains, as previously reported [11]. In the C57/BL6 pigmented mouse their density is very low in the superior retina (most of the dorsal retina is purple, i.e. between 0 and 2,250 cones/mm^2^), while in the ventro-nasal retina they reach densities above 18,000 cones/mm^2^. In the albino Swiss mouse there is also an S-opsin dorso-ventral gradient where the highest densities are found ventro-nasally. However, in this strain the dorsal retina is also highly populated by S-opsin^+^cones, reaching densities between 13,501 and 15,750 cones/mm^2^ (orange).

Finally, the distribution of all-cones is a mixture of the single opsin distributions and, in general terms, similar in both strains. Cones are distributed across the retina, with a gradient of increasing density from the dorsal to the ventral retina and a tendency to higher densities in the nasal retina.

### L-opsin^+^cones and RGCs

**Figure 5:**
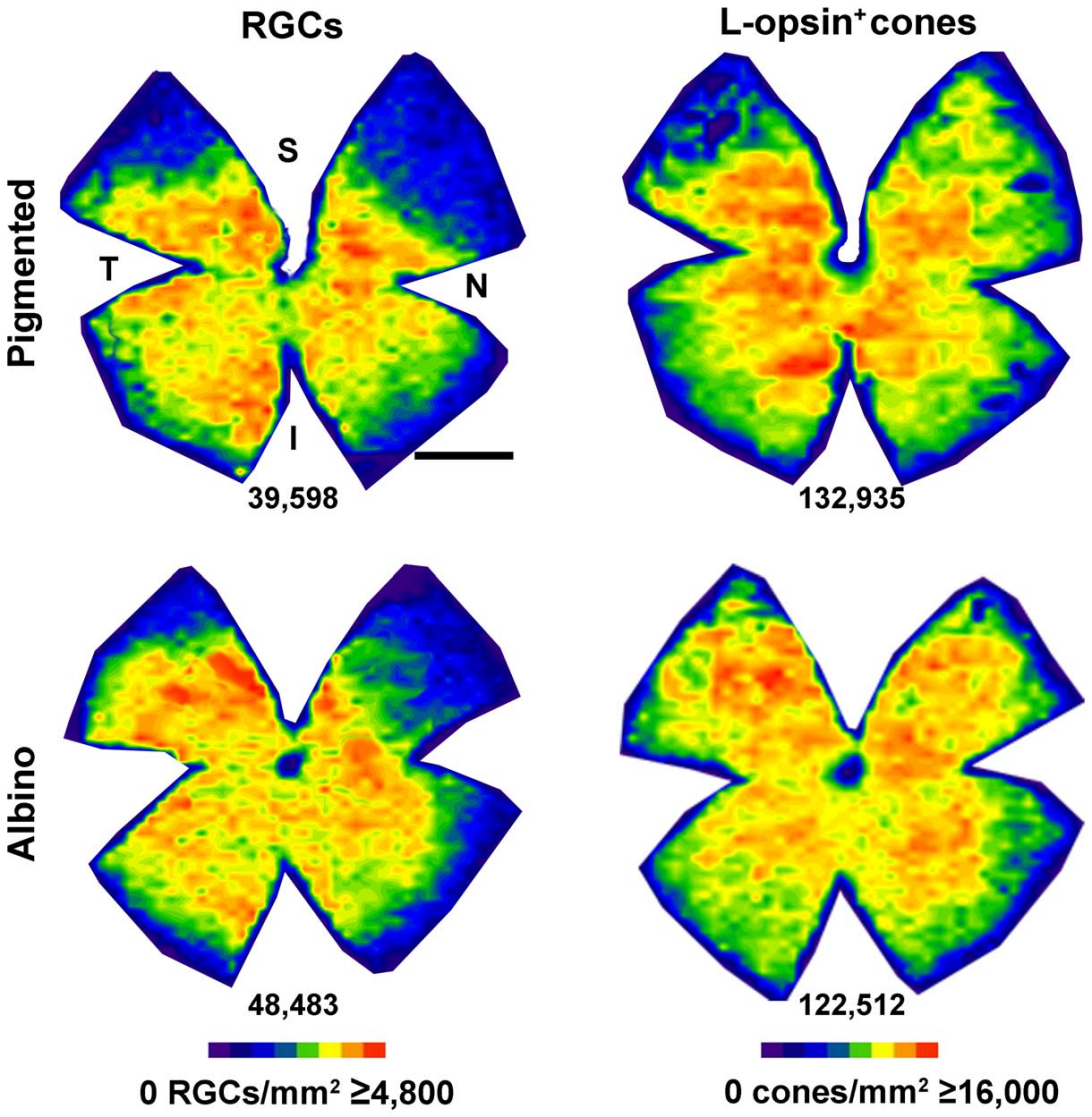
Distribution of RGCs and L-opsin^+^cones. Isodensity maps showing the distribution RGCs and L-opsin^+^cones in the same retina of a pigmented (first row) and an albino (second row) mouse. Below each map is shown the number of cells quantified in its corresponding retina. Colour scale density code is at the bottom of the figure. S: superior, N: nasal, I: inferior, T: temporal. Bar: 1 mm.

We have previously shown that in rat there is a parallel distribution of L-cones and RGCs [12], reinforcing the notion of a visual streak placed above the optic nerve where L-cones and RGCs are densest while the S-cones are sparsest [12], [25], . We wanted to study whether the same co-distribution was observed in mouse. Thus, Brn3a and L-opsin were double immunodetected in retinas from both mouse strains. After quantification of the total number of RGCs and cones (mean number±SD of RGCs and L-opsin^+^cones: 41,860±1,579 and 137,009±5,802 in pigmented mice (n = 6), and 51,205±1,977 and 124,497±14,532 in albinos (n = 4), respectively), isodensity maps were generated (**Figure 5**). In these maps it is observed that in both strains RGCs and L-cones are denser in the central retina thinning out towards the periphery.

### Sampling for accurate inference of total cone numbers

**Figure 6:**
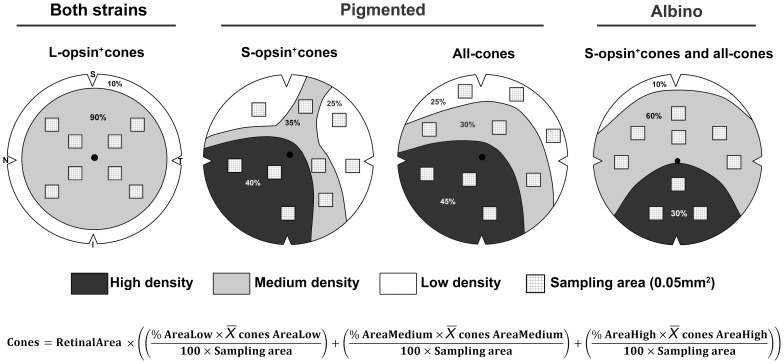
How to sample the mouse retina to accurately infer each cone population. Schematic drawings of a right retina showing the minimum number of sampling areas of 0.05^2^ needed to manually quantify and infer the total cone population in both strains. In each drawing are shown the cone density regions wherefrom the samples should be taken. The inference of the total cone population can be calculated following the mathematical formula at the bottom of the figure, where the retinal area should be previously measured in mm^2^, % of area low is 25, % of area medium is 30–90, % of area high is 30–45 (depending on the cone population, see drawings), sampling area is 0.05 mm^2^ and 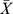 is the mean number of cones per density area (i.e. average of cones in the samples taken within each density region). S: superior, N: nasal, I: inferior, T: temporal.

Automated quantification has several advantages among which are: saving time, accuracy, objectivity and, of course, knowing the whole population of a given cell in a determined paradigm such as health, disease or neuroprotection. But because these routines are not available in all laboratories, we next purposed to design a sampling method to calculate reliably the total cone population after manual quantification of the samples (**Figure 6**).

Although for the albino mouse, immunodetection of the S-opsin would suffice to identify the whole cone population, we also identified the sampling areas of L-opsin^+^cones because in some experimental paradigms (e.g. phototoxicity to red light) these might be specifically affected and thus studied without taking into account the pool of genuine S-opsin^+^cones.

First, based on the isodensity maps (**Figure 4**) we determined the density areas where samples should be taken for each cone type and strain. For this, all isodensity maps were analyzed. Then, the percent of the retinal area occupied by each density region was calculated (**Figure 6**). For each density area, 3–8 samples (0.05 mm^2^ each) were acquired and quantified. Of note, although we pictured the samples in particular places according with the pattern of high, medium and low density shown by the isodensity maps, samples may be taken anywhere within their density area. In addition, the sample size is smaller than a capturing frame for practical reasons; it is achievable to count manually cones in 0.05 mm^2^, while in a whole frame (0.2186 mm^2^) the abundance of cones would make manual counts too cumbersome. However, the sample size can be incremented at will; because of this the denominator in the formula shown in **Figure 6** is 100×sampling area, instead of 5 (100×0.05 mm^2^).

Note that to quantify the S-opsin^+^cones and all-cones in the albino mouse and the L-opsin^+^cones in both strains, the low density area is not sampled. Besides, in the latter, the medium and high densities have been joined into one to simplify the method, since the results are accurate enough and similar to the first approximation we did considering medium and high densities separately.

Finally, the total cone population was inferred by extrapolating the mean counts of the samples to their density areas and these to the retinal area, using the formula in **Figure 6**. Evidently, for the L-opsin^+^cones and the S-opsin^+^cones, and all-cones in albino, the formula will be reduced to the sampled density area. For instance, to infer the population of L-opsin^+^cones the formula would be:
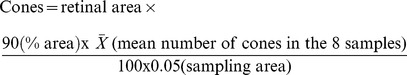



The number±SD of inferred cones in the pigmented mouse was: 138,440±11,253 L-opsin^+^cones, 121,342±6,650 S-opsin^+^cones, and 182,194±8,215 all-cones, and in the albino mouse was: 123,147±16,146 L-opsin^+^cones, 149,128±24,281 S-opsin^+^cones, and 158,253±10,552 all-cones. Because these values do not differ from those obtained after automated quantification (see **table 1**), our sampling method is accurate and valid to analyze manually the cone population and improvable by increasing the number of samples per density area and/or their size.

## Discussion

There are several articles reporting the density and number of cone photoreceptors in mice (see below). However, this is the first work addressing in detail the total number and distribution of the different spectral cone types. This is important as it lays the basis for further research in cone degenerative paradigms, which is currently a topic of extensive research. In general, mutants [22] or transgenic [15] mice have a pigmented background whereas studies on phototoxic cone death are carried out in albino mice [32].

In the present study we have taken advantage of our experience in automated quantification of the entire population of RGCs [23], [25]–[28] as well as cone-outer segments in whole mounted rat retinas [12], to design routines to determine accurately the total number of all-cones and of cones that express the L- or S- opsin in mice. Moreover, we also describe the retinal topography of each of these two cone populations, and of all-cones. These experiments are based on the immunohistofluorescence identification of the opsins located in the outer segment of the photoreceptor, a method that was validated for the albino and pigmented adult rat retina [12] and has been validated here for the albino Swiss and the pigmented C57/BL6 mouse strains. Thus, caution should be taken when interpreting the data in situations where injury may alter the expression pattern of these opsins.

Albino mice and rats suffer from impaired visual acuity, defects in the optokinetic nystagmus, lower numbers of rods as well as of rhodopsin expression, and have a lower ratio of ipsilateral projections from the RGCs than pigmented animals [26], [37]–[40]. In agreement with our data, Whitney et al [13] observed that albino A/J mice had fewer cones than pigmented B6/J ones. Interestingly, in their quantitative trait locus study of the AxB and BxA RI strains they discovered that the differences in the total number of cones were not linked to albinism. However, changes in the retinal topography of S-opsin^+^cones in the house mouse are associated with the lack of melanin, as indicated by our results and others' [8], [11]. Here we show that within the smaller population of total cones found in the Swiss albino mice, the percentage of dual cones (i.e. more cones expressing the S-opsin) increases approximately to 73% at the expense of genuine L-cones.

With respect to this, there are species-specific patterns. In rat, although the number of S-cones is significantly higher in albino (15% of the total cone population) than in pigmented (10%), their topography is similar. Furthermore, we have shown that in both rat strains dual cones amount to 3% of the total cone population and that they are found predominantly in the far retinal periphery, indicating that albinism in rat does not affect the number of dual cones nor the cone topography [12]. In the deer mouse [20], it has been shown that cone densities in wild type and albino animals are similar, but while in pigmented animals dual cones are rare and there is a dorso-ventral gradient of S-opsin, in albino retinas most cones are dual and this gradient is lost. In the present studies in the house mouse we show that pigmented animals have a higher net number of cones, out of which approximately 40% are dual. In albino mice, as occurs in the deer mouse, the increase in the number of dual cones results in a change of the S-opsin topography. Why is there such a shift in opsin expression? It has been postulated that in albino mice, the balance of appropriate transcription factors needed to decrease the S-opsin expression in the dorsal retina is impaired, thus resulting in a higher number of dual cones [6], [8], [20].

Genuine-S cones are rare and their published percentages in pigmented mice range from 3 to 20% [4], [6], [8],[41]. Our estimation from the total quantification of S-opsin^+^, L-opsin^+^, and all- cones give a percentage of 26% of genuine-S cones. Nikonov et al [41] studied the electrophysiological response of individual cones in retinal slices and observed that only 1 out of 30 S-opsin^+^cones behaved as a genuine-S cone (i.e. around 3%). Röhlich et al [6] counted genuine-S cones only in the transition zone between the dorsal and ventral retina. Haverkamp et al [4] analyzed sampling areas and found 3–5% genuine-S cones in the dorsal retina and between 8 and 20% in the ventral, these data are closer to ours. Nevertheless, there are still differences between our data and these previous reports that may be explained by the different approaches used. However, we cannot rule out that some S-opsin^+^cones may express low levels of L-opsin which would fall below the sensitivity of our epifluorescence technique.

The diversity of photoreceptor arrangements in mammals has been selected evolutionarily for each lifestyle and habitat (reviewed in [42]). In mouse, dual cones broaden the spectral range to which a cone is sensitive, allowing a better vision in varied spectral compositions of ambient light [10]. Furthermore, the S-opsin in the ventral retina encodes preferentially dark contrast and so the S-opsin^+^cones could be the sky sensors for birds of prey. According to our topographic maps albino mice have, as the pigmented ones, the highest densities of S-opsin^+^cones in the ventral retina. The L-opsin in the dorsal retina is used to see the ground [43], and its distribution is similar between both strains. Thus, the mouse retina has evolved to finely perceive its natural environment.

The maximum densities of S-opsin^+^cones in pigmented mice are found in the naso-ventral retina, this may be related to two important findings: i/ S-opsin^+^cones contribute to non image forming vision responses, supporting sustained activation of the pretectal olivary nucleus (central origin of the pupillary light reflex) [44] and, ii/ in the nasal retina there is a discrete plexus of melanopsin-expressing RGCs with direct retino-ciliary projections whose suggested role is to participate in the intrinsic pupillary light reflex by sending signals from the retina to the iris via the ciliary body [45].

It has been previously reported [8], [15], [16], [18] that cones that express the L opsin, are preferentially found in the dorsal retina. Our topographical analysis shows that this is not so. In both mouse strains L-opsin^+^cones are found across the retina with a density gradient declining from the centre to the periphery. Nevertheless, in the pigmented strain genuine-L cones are located in the dorsal retina where there are few S-opsin^+^cones.

In spite of the difference in the topography of S-opsin^+^cones between both strains the distribution of all-cones is practically the same, although in the albino strain densities are slightly lower due to its lower number of cones. In contrast to previous reports [8], [46] we show that the topography of all-cones is not even, rather there is an increasing density gradient from the dorsal to the ventral retina.

The total number of cones (all-cones) quantified here in the albino Swiss and pigmented C57/BL6 strains is, respectively, 153,043 and 182,601. The latter is in agreement with the number estimated by other groups [8], [46] but slighty lower than those estimated by Withney et al [13], a discrepancy that could be explained by the quantificaton method: they estimated the total cone number from samples while here the whole cone population was counted.

Taking into account the total number of cones in this work and that the total number of rods in C57 mice has been reported to be of around 6.4 millions [46], then cones represent approximately 2.8% of the total photoreceptor population, a proportion that is also in agreement with previous studies [46], [47]. In albino mice the number of rods is reduced by 25% [38], which gives a total of 4.8 million rods. So, based on our data, in the albino Swiss mice cones are approximately 3.1% of the total photoreceptor population.

Regarding the numbers of S- or L- opsin^+^cones, mean or regional densities from albino or pigmented mice have been reported in several studies [8], [9], [17], [18], [48]. Published densities of L-opsin^+^cones in pigmented mouse range from 8,141 to 12,000 cells/mm^2^ [8], [9]. Here we show that their mean density is 9,420 cells/mm^2^. Cone isodensity maps in albino mice were first published by Szel et al. [18]. Szel and colleagues reported that the maximum density of L-cones was 4,000 cells/mm^2^ and of S-cones 11,000 cells/mm^2^. Our maximum densities are higher for both populations (L-opsin^+^cones 13,879 cells/mm^2^, and S-opsin^+^cones 17,553 cells/mm^2^). The dorsal/ventral ratio of S-cones in albino and pigmented animals reported by [8] was 1∶4 and 1∶30, respectively. In our present studies we present similar ratios for albino (1∶3.2) but for pigmented mice we observe a threefold increase (1∶96). These differences may be explained by strain differences and/or by the quantification approach.

In rat, the topography of L-cones parallels the spatial distribution of RGCs [12]. In mouse, both topographies are comparable across the retina except for the dorso-nasal quadrant, where the density of RGCs is low and of L-opsin^+^cones is high. In rat, however, the density of L-cones in this retinal quadrant is low and thus the distribution of RGCs and L-cones is analogous all over the retina. Thus, in mouse, RGCs and L-opsin^+^cones have similar, but not perfectly matched, distributions. Finally, because the highest RGC and cone densities are found above the optic nerve forming a streak in rats and in the central retina around the optic nerve in mouse, we would like to suggest here that experiments aimed at restoring vision by cell transplantation or gene therapy in RGC or cone degenerative paradigms, should be focused on these retinal regions.

Sampling standard areas of the retina for manual counting has been a classical method to calculate among other, retinal ganglion cells [49]–[51], microglial cells [52] and cone [8], [13], [14], [16]–[18] densities. Furthermore, the inference of the total RGC population after manual sampling and counting in specific retinal areas has been proved to be a reliable method in rat and mouse [23], [29], [33]. Based on the isodensity maps of each cone population we have designed a method that allows inferring the total number of each cone type after sampling and quantifying manually specific retinal areas. The method proposed may be useful to assess the cone population when automated routines are not available. Although it should be noticed that this sampling method is only valid for those strains whose cone topographies resemble the ones shown here.

In summary, we show here the first comprehensive analysis of cone photoreceptor numbers and retinal distribution in C57/BL6 and Swiss inbred mice. These data may provide the baseline for future work aimed to study cone degeneration and neuroprotection in these two strains.

## References

[1] Jacobs GH, Williams GA, Fenwick JA (2004) Influence of cone pigment coexpression on spectral sensitivity and color vision in the mouse. Vision Res 44: 1615–1622. 15135998 10.1016/j.visres.2004.01.016

[2] Lyubarsky AL, Falsini B, Pennesi ME, Valentini P, Pugh EN Jr (1999) UV- and midwave-sensitive cone-driven retinal responses of the mouse: a possible phenotype for coexpression of cone photopigments. J Neurosci 19: 442–455. 9870972 10.1523/JNEUROSCI.19-01-00442.1999 PMC6782392

[3] Yokoyama S, Radlwimmer FB, Kawamura S (1998) Regeneration of ultraviolet pigments of vertebrates. FEBS Lett 423: 155–158. 9512349 10.1016/s0014-5793(98)00086-6

[4] Haverkamp S, Wassle H, Duebel J, Kuner T, Augustine GJ, et al (2005) The primordial, blue-cone color system of the mouse retina. J Neurosci 25: 5438–5445. 15930394 10.1523/JNEUROSCI.1117-05.2005 PMC6725002

[5] Li W, DeVries SH (2006) Bipolar cell pathways for color and luminance vision in a dichromatic mammalian retina. Nat Neurosci 9: 669–675. 16617341 10.1038/nn1686

[6] Röhlich P, van Veen T, Szel A (1994) Two different visual pigments in one retinal cone cell. Neuron 13: 1159–1166 0896-6273(94)90053-1 [pii]. 7946352 10.1016/0896-6273(94)90053-1

[7] Lukats A, Szabo A, Röhlich P, Vigh B, Szel A (2005) Photopigment coexpression in mammals: comparative and developmental aspects. Histol Histopathol 20: 551–574. 15736061 10.14670/HH-20.551

[8] Applebury ML, Antoch MP, Baxter LC, Chun LL, Falk JD, et al (2000) The murine cone photoreceptor: a single cone type expresses both S and M opsins with retinal spatial patterning. Neuron 27: 513–523. 11055434 10.1016/s0896-6273(00)00062-3

[9] Williams GA, Jacobs GH (2007) Cone-based vision in the aging mouse. Vision Res 47: 2037–2046. 17509638 10.1016/j.visres.2007.03.023 PMC2049007

[10] Chang L, Breuninger T, Euler T (2013) Chromatic coding from cone-type unselective circuits in the mouse retina. Neuron 77: 559–571. 23395380 10.1016/j.neuron.2012.12.012

[11] Jelcick AS, Yuan Y, Leehy BD, Cox LC, Silveira AC, et al (2011) Genetic variations strongly influence phenotypic outcome in the mouse retina. PLoS One 6: e21858. 21779340 10.1371/journal.pone.0021858 PMC3136482

[12] Ortin-Martinez A, Jimenez-Lopez M, Nadal-Nicolas FM, Salinas-Navarro M, Alarcon-Martinez L, et al (2010) Automated quantification and topographical distribution of the whole population of S- and L-cones in adult albino and pigmented rats. Invest Ophthalmol Vis Sci 51: 3171–3183. 20071667 10.1167/iovs.09-4861

[13] Whitney IE, Raven MA, Lu L, Williams RW, Reese BE (2011) A QTL on chromosome 10 modulates cone photoreceptor number in the mouse retina. Invest Ophthalmol Vis Sci 52: 3228–3236. 21330668 10.1167/iovs.10-6693 PMC3109025

[14] Fei Y (2003) Development of the cone photoreceptor mosaic in the mouse retina revealed by fluorescent cones in transgenic mice. Mol Vis 9: 31–42. 12592228

[15] Kuny S, Filion MA, Suh M, Gaillard F, Sauve Y (2013) Long-term retinal cone survival and delayed alteration of the cone mosaic in a transgenic mouse model of Stargardt-like dystrophy (STGD3). Invest Ophthalmol Vis Sci 10.1167/iovs.13-13457 24334447

[16] Szel A, Röhlich P, Caffe AR, Juliusson B, Aguirre G, et al (1992) Unique topographic separation of two spectral classes of cones in the mouse retina. J Comp Neurol 325: 327–342. 1447405 10.1002/cne.903250302

[17] Szel A, Röhlich P, Mieziewska K, Aguirre G, van Veen T (1993) Spatial and temporal differences between the expression of short- and middle-wave sensitive cone pigments in the mouse retina: a developmental study. J Comp Neurol 331: 564–577. 8509512 10.1002/cne.903310411

[18] Szel A, Csorba G, Caffe AR, Szel G, Röhlich P, et al (1994) Different patterns of retinal cone topography in two genera of rodents, Mus and Apodemus. Cell Tissue Res 276: 143–150. 8187156 10.1007/BF00354793

[19] Neitz M, Neitz J (2001) The uncommon retina of the common house mouse. Trends Neurosci 24: 248–250. 11311361 10.1016/s0166-2236(00)01773-2

[20] Arbogast P, Glösmann M, Peichl L (2013) Retinal Cone Photoreceptors of the Deer Mouse Peromyscus maniculatus: Development, Topography, Opsin Expression and Spectral Tuning. PLoS One 8: e80910. 24260509 10.1371/journal.pone.0080910 PMC3829927

[21] Huberman AD, Niell CM (2011) What can mice tell us about how vision works? Trends Neurosci 34: 464–473. 21840069 10.1016/j.tins.2011.07.002 PMC3371366

[22] Chang B, Hawes NL, Hurd RE, Davisson MT, Nusinowitz S, et al (2002) Retinal degeneration mutants in the mouse. Vision Res 42: 517–525. 11853768 10.1016/s0042-6989(01)00146-8

[23] Galindo-Romero C, Aviles-Trigueros M, Jimenez-Lopez M, Valiente-Soriano FJ, Salinas-Navarro M, et al (2011) Axotomy-induced retinal ganglion cell death in adult mice: quantitative and topographic time course analyses. Exp Eye Res 92: 377–387. 21354138 10.1016/j.exer.2011.02.008

[24] Galindo-Romero C, Jimenez-Lopez M, Garcia-Ayuso D, Salinas-Navarro M, Nadal-Nicolas FM, et al (2013) Number and spatial distribution of intrinsically photosensitive retinal ganglion cells in the adult albino rat. Exp Eye Res 108: 84–93. 23295345 10.1016/j.exer.2012.12.010

[25] Nadal-Nicolas FM, Jimenez-Lopez M, Sobrado-Calvo P, Nieto-Lopez L, Canovas-Martinez I, et al (2009) Brn3a as a marker of retinal ganglion cells: Qualitative and quantitative time course studies in naive and optic nerve injured retinas. Invest Ophthalmol Vis Sci 50 8: 3860–8. 19264888 10.1167/iovs.08-3267

[26] Nadal-Nicolas FM, Jimenez-Lopez M, Salinas-Navarro M, Sobrado-Calvo P, Alburquerque-Bejar JJ, et al (2012) Whole number, distribution and co-expression of Brn3 transcription factors in retinal ganglion cells of adult albino and pigmented rats. PLoS One 7: e49830. 23166779 10.1371/journal.pone.0049830 PMC3500320

[27] Salinas-Navarro M, Mayor-Torroglosa S, Jimenez-Lopez M, Aviles-Trigueros M, Holmes TM, et al (2009) A computerized analysis of the entire retinal ganglion cell population and its spatial distribution in adult rats. Vision Res 49: 115–126. 18952118 10.1016/j.visres.2008.09.029

[28] Salinas-Navarro M, Jimenez-Lopez M, Valiente-Soriano FJ, Alarcon-Martinez L, Aviles-Trigueros M, et al (2009) Retinal ganglion cell population in adult albino and pigmented mice: a computerized analysis of the entire population and its spatial distribution. Vision Res 49: 637–647. 19948111 10.1016/j.visres.2009.01.010

[29] Galindo-Romero C, Valiente-Soriano FJ, Jimenez-Lopez M, Garcia-Ayuso D, Villegas-Perez MP, et al (2013) Effect of brain-derived neurotrophic factor on mouse axotomized retinal ganglion cells and phagocytic microglia. Invest Ophthalmol Vis Sci 54: 974–985. 23307961 10.1167/iovs.12-11207

[30] Garcia-Ayuso D, Salinas-Navarro M, Nadal-Nicolas FM, Ortin-Martinez A, Agudo-Barriuso M, et al (2013) Sectorial loss of retinal ganglion cells in inherited photoreceptor degeneration is due to RGC death. Br J Ophthalmol 98 3: 396–401. 24326325 10.1136/bjophthalmol-2013-303958 PMC3933073

[31] Garcia-Ayuso D, Ortin-Martinez A, Jimenez-Lopez M, Galindo-Romero C, Cuenca N, et al (2013) Changes in the photoreceptor mosaic of P23H-1 rats during retinal degeneration: implications for rod-cone dependent survival. Invest Ophthalmol Vis Sci 54: 5888–5900. 23908186 10.1167/iovs.13-12643

[32] Montalban-Soler L, Alarcon-Martinez L, Jimenez-Lopez M, Salinas-Navarro M, Galindo-Romero C, et al (2012) Retinal compensatory changes after light damage in albino mice. Mol Vis 18: 675–693. 22509098 PMC3325904

[33] Sanchez-Migallon MC, Nadal-Nicolas FM, Jimenez-Lopez M, Sobrado-Calvo P, Vidal-Sanz M, et al (2011) Brain derived neurotrophic factor maintains Brn3a expression in axotomized rat retinal ganglion cells. Exp Eye Res 92: 260–267. 21315070 10.1016/j.exer.2011.02.001

[34] Vidal-Sanz M, Salinas-Navarro M, Nadal-Nicolas FM, Alarcon-Martinez L, Valiente-Soriano FJ, et al (2012) Understanding glaucomatous damage: anatomical and functional data from ocular hypertensive rodent retinas. Prog Retin Eye Res 31: 1–27. 21946033 10.1016/j.preteyeres.2011.08.001

[35] Ortin-Martinez A, Nadal-Nicolas F, Jimenez-Lopez M, Villegas-Perez MP, Vidal-Sanz M, et al (2013) Total number and spatial distribution of mouse cones. Ophthalmic Research 50: 40.

[36] Vidal-Sanz M, Ortin-Martinez A, Nadal-Nicolas FM, Jimenez-Lopez M, Alburquerque-Bejar JJ, et al (2014) Differences in cone type proportions and distribution between pigmented and albino strains of adult mice. Invest Ophthalmol Vis Sci 55 E-Abstract 4366.

[37] Balkema GW, Dräger UC (1991) Impaired visual thresholds in hypopigmented animals. Vis Neurosci 6: 577–585. 1883762 10.1017/s095252380000256x

[38] Donatien P, Jeffery G (2002) Correlation between rod photoreceptor numbers and levels of ocular pigmentation. Invest Ophthalmol Vis Sci 43: 1198–1203. 11923266

[39] Dräger UC, Balkema GW (1987) Does melanin do more than protect from light? Neurosci Res Suppl 6: S75–S86. 10.1016/0921-8696(87)90009-0 3317148

[40] Prusky GT, Harker KT, Douglas RM, Whishaw IQ (2002) Variation in visual acuity within pigmented, and between pigmented and albino rat strains. Behav Brain Res 136: 339–348. 12429395 10.1016/s0166-4328(02)00126-2

[41] Nikonov SS, Kholodenko R, Lem J, Pugh EN Jr (2006) Physiological features of the S- and M-cone photoreceptors of wild-type mice from single-cell recordings. J Gen Physiol 127: 359–374. 16567464 10.1085/jgp.200609490 PMC2151510

[42] Peichl L (2005) Diversity of mammalian photoreceptor properties: adaptations to habitat and lifestyle? Anat Rec A Discov Mol Cell Evol Biol 287: 1001–1012. 16200646 10.1002/ar.a.20262

[43] Baden T, Schubert T, Chang L, Wei T, Zaichuk M, et al (2013) A Tale of Two Retinal Domains: Near-Optimal Sampling of Achromatic Contrasts in Natural Scenes through Asymmetric Photoreceptor Distribution. Neuron 80: 1206–1217. 24314730 10.1016/j.neuron.2013.09.030

[44] Allen AE, Brown TM, Lucas RJ (2011) A distinct contribution of short-wavelength-sensitive cones to light-evoked activity in the mouse pretectal olivary nucleus. J Neurosci 31: 16833–16843. 22090509 10.1523/JNEUROSCI.2505-11.2011 PMC3245852

[45] Semo M, Gias C, Ahmado A, Vugler A (2014) A role for the ciliary marginal zone in the melanopsin-dependent intrinsic pupillary light reflex. Exp Eye Res 119: 8–18. 24316157 10.1016/j.exer.2013.11.013

[46] Jeon CJ, Strettoi E, Masland RH (1998) The major cell populations of the mouse retina. J Neurosci 18: 8936–8946. 9786999 10.1523/JNEUROSCI.18-21-08936.1998 PMC6793518

[47] Carter-Dawson LD, LaVail MM (1979) Rods and cones in the mouse retina. J Comp Neurol 188: 245–272. 500858 10.1002/cne.901880204

[48] Szel A, Diamantstein T, Röhlich P (1988) Identification of the blue-sensitive cones in the mammalian retina by anti-visual pigment antibody. J Comp Neurol 273: 593–602. 3209737 10.1002/cne.902730413

[49] Lindqvist N, Peinado-Ramonn P, Vidal-Sanz M, Hallbook F (2004) GDNF, Ret, GFRalpha1 and 2 in the adult rat retino-tectal system after optic nerve transection. Exp Neurol 187: 487–499. 15144875 10.1016/j.expneurol.2004.02.002

[50] Villegas-Perez MP, Vidal-Sanz M, Lund RD (1996) Mechanism of retinal ganglion cell loss in inherited retinal dystrophy. Neuroreport 7: 1995–1999. 8905711 10.1097/00001756-199608120-00028

[51] Wang S, Villegas-Perez MP, Holmes T, Lawrence JM, Vidal-Sanz M, et al (2003) Evolving neurovascular relationships in the RCS rat with age. Curr Eye Res 27: 183–196. 14562184 10.1076/ceyr.27.3.183.16053

[52] Sobrado-Calvo P, Vidal-Sanz M, Villegas-Perez MP (2007) Rat retinal microglial cells under normal conditions, after optic nerve section, and after optic nerve section and intravitreal injection of trophic factors or macrophage inhibitory factor. J Comp Neurol 501: 866–878. 17311318 10.1002/cne.21279

